# Biological Pathotyping of Newcastle Disease Viruses in Sudan 2008–2013

**DOI:** 10.1155/2014/209357

**Published:** 2014-11-26

**Authors:** Egbal Sidahmed Abdelrahim Bilal, Iman Mohammed Elnasri, Aymen Mohamed Alhassan, Khalda Abdelaziz Khalifa, Jedddha Ibrahim Elhag, Selma Osman Ahmed

**Affiliations:** Veterinary Research Institute, P.O. Box 8067, Khartoum, Sudan

## Abstract

The biological properties and pathogenicity of seven Newcastle disease virus field isolates were studied. These isolates were recovered from different outbreaks in Sudan (5 from chickens and 2 from pigeons) during 2008–2013. Based on intracerebral pathogenicity index, four NDV isolates were characterized as velogenic (their ICPI ranged 2.0–1.6) and three isolates were characterized as mesogenic (ICPI ranged 1.2–1.3). The mean death time for all isolates ranged from 54 to 76.8 hours. The elution time of the viruses from chicken erythrocytes and the ability to haemagglutinate mammalian red blood cells differed considerably in their reactions.

## 1. Introduction

Newcastle disease (ND) is still considered as an economically important disease which is highly contagious infection for many avian species. ND is caused by virulent strains of avian paramyxovirus type 1 (APMV-1) of the genus* Avulavirus* belonging to the family Paramyxoviridae [1]. According to the World Organization for Animal Health (OIE, 2012), ND is an OIE notifiable disease when it meets certain criteria of virulence.

Newcastle disease affects a wide range of domestic and wild avian species; however, the severity of the disease varies greatly, spanning from peracute disease with almost 100% mortality to subclinical disease with no lesions. Such variability makes it impossible to pinpoint ND as a single clinicopathologic entity [2]. Based on severity of clinical disease, the strains of NDV were originally classified into 3 groups based on their virulence such as lentogenic, mesogenic, and velogenic. Lentogenic strains, especially in adult chickens, may cause minimal or no clinical signs. However, the disease produced by mesogenic strains may cause mortality that can reach 25% and those by in velogenic strains may reach up to 100% [3] Differences in virulence of the virus occur during any disease outbreak, so determining virulence is essential for the effective control of the disease.

ND was first reported in Sudan in 1951 [4] and still constituted a major problem facing the poultry industry. Newcastle disease virus (NDV) exhibits a wide range of pathogenicity and virulence. Assessment of the virulence of NDV is necessary in order to limit the outbreaks and to minimize their impact. So the aim of this study was to evaluate the pathogenicity of some NDV field strains isolated 2008–2013.

## 2. Materials and Methods

### 2.1. Viruses

Seven NDV viruses were isolated from different hosts, including chickens (layers and broilers) and pigeons from several regions in Sudan during 2008 to 2013 from cases submitted for diagnosis to the Department of Avian Diseases and Diagnosis, Veterinary Research Institute, Khartoum, Sudan. The information concerning the origin of virus outbreaks, the year of isolation, breed, type, and age of birds involved is presented in Table 1. Mortality during the outbreak varied from 90% in young chickens to less than 2% in hens; however, egg production dropped from 90 to 40%. Clinical signs of the disease were similar among most poultry farms including dyspnea, nervous, respiratory signs, diarrhea, and cyanosis of the combs.

### 2.2. Chicks and Embryonated Egg

Nine-to-eleven-day-old embryonated eggs (Bovan) and chicks (cockerels) were provided by the Coral Farm Hatcheries. Before use the chicks were checked for NDV antibodies and were found negative.

### 2.3. Virus Isolation and Identification

Filtrates of processed tissues from the trachea, lung, brain, spleen, and cecal tonsils from different hosts were used to inoculate 9–11-day embryonated eggs for virus isolation via allantoic route and the NDV was identified by means of HA and HI test using standards procedures [1]. Virus stocks grown in allantoic fluids were stored at −20°C until being used.

### 2.4. Haemagglutination of Chicken and Mammalian Erythrocytes

The test was performed in V-shaped microtitre plates. Volumes of 25 *μ*L of PBS were placed in each well of the plate followed by 25 *μ*L of allantoic fluid of virus isolate in each well in the first row. After 2 fold dilutions were carried out in subsequent rows, 25 *μ*L of 1% suspension of chickens, cattle, sheep, and equine RBCs was added to each well. After 30 minute of incubation at room temperature, the reciprocal of the highest dilution that produced positive HA was considered as positive virus titre.

### 2.5. Haemagglutination Elution Test

The test was done following the procedures described previously [5, 6]. A standard HA test was performed with chicken RBCs in WHO haemagglutination plates using 500 *μ*L volumes. The test was incubated at 4°C for 1 hour before the HA titre was calculated. After gentle mixing and further incubation, the HA titre was also recorded at 24 and 30 hours.

### 2.6. Biological Pathogenicity Assessment

#### 2.6.1. Mean Death Time of the Minimum Lethal Dose (MDT/MLD)

MDT was performed in 9-10-day-old embryonated egg. Serial tenfold dilutions of allantoic fluid (AF) of each virus isolate were prepared and 0.1 mL of the dilutions (10^−5^, 10^−8^, and 10^−9^) was inoculated into the allantoic cavity using 5 eggs per dilution. The highest dilution at which all embryos died soon was considered as mean lethal dose (MLD) and the MDT/MLD was calculated as described [7].

#### 2.6.2. Intracerebral Pathogenicity Index (ICPI) Assessment

Pathogenicity of NDV isolates was assessed by using the standard intracerebral pathogenicity index (ICPI) test. Briefly, ten-one-day-old chicks were inoculated intracerebrally with 0.1 mL of a 1 : 10 dilution of allantoic fluid of each virus isolate. Ten chicks were kept as uninoculated control. All chicks were monitored during an eight-day observation period and scored as normal (0), sick or paralyzed (1), and dead (2). Total scores were determined, and the mean daily score was calculated to obtain the ICPI as described [8].

## 3. Results

### 3.1. Virus Isolation

Following inoculation of embryonated chicken eggs with suspensions from organs of diseased birds, embryo died 2-3 days after inoculation for the Pmed/010, Bhalf/011, Bso/013, and Lkab/013 and 4-5 days for LNy/08, LNy/09, and Pkam/09 NDV isolates.

The harvested allantoic fluids from dead eggs agglutinated red blood cells of chickens and this agglutination was inhibited by addition of specific NDV antiserum.

### 3.2. Haemagglutination of Chicken and Mammalian Erythrocytes

Table 2 shows the HA pattern of field NDV isolates. The Pkam/09, Bso/013, and Lkab/013 agglutinated equine, sheep, and bovine RBCs while LNy/09 agglutinated only the equine RBCs. The LNy/08, Pmed/010, and Bhalf/011 did not agglutinate any of the mammalian species RBCs.

### 3.3. Haemagglutination Elution Test

The haemagglutination elution of the NDV field isolate recorded after 1, 24, and 30 hours was illustrated in Table 3. There is a difference in HA titre recorded in 24 and 30 hours of all isolates.

### 3.4. Pathogenicity Test

#### 3.4.1. Mean Death Time of the Minimum Lethal Dose (MDT/MLD)

The MDT of the NDV isolates was presented in Table 4. MDT ranged from 54–76.8 hours for all isolates.

The ICPI of the LNy/08, LNy/09, and Pkam/09 was 1.3, 1.2, and 1.3 which can be classified as mesogenic strains. The ICPI of the Pmed/010, Bhalf/011, Bso/013, and Lkab/013 was 1.8, 2.0, 1.6, and 1.6, respectively, which classified as velogenic NDV strains.

## 4. Discussion

Newcastle disease (ND) is a serious and commonly fatal viral poultry disease, which is present all over the world. Viral characterization using the pathogenicity test or molecular typing is essential, as the importance and impact of a given NDV isolate are directly related to its virulence.

In the present study NDV isolates were from outbreaks of different locations and five isolates were recovered from chickens while two were from pigeons.

The ability of NDV to agglutinate red blood cells (RBCs) is due to the binding of the HN protein to receptors on the surface of the RBCs. Chicken RBCs are usually used in HA tests but NDV will cause agglutination of all amphibian, reptilian, and avian cells. The ability of NDV strains to agglutinate cattle, goat, sheep, swine, and horse RBCs varied among various strains [9]. Ibu et al. (2009) [10] applied the haemagglutinability tests using mammalian erythrocytes as a tool for strain differentiation. Here among the field isolates tested, three isolates agglutinated mammalian RBCs. It is noticed that there is a difference in hemagglutination patterns which could not be correlated to the virulence of the NDV isolates. This result was similar to previous research [11] and disagrees with the suggestion of others [12] that the pattern of agglutination of some NDV strains may be related to the degree of virulence that more virulent strains appear to show less agglutinability of certain mammalian red blood cells than the less virulent ones.

Elution time could form a basis for rough characterization of isolates of Newcastle disease virus [5, 6, 13, 14]. The NDV isolates used in this study were slow HA eluters. Their elution reactions appear not to be correlated to their virulence.

Previously no relationship was found between haemagglutination elution patterns and other properties of the same strain [5, 6].

The in vivo assessment of virus virulence is based on the ICPI test, which is considered as the most sensitive and widely used test for measuring virulence [2]. In the present study, based on ICPI, the results indicated that four NDV isolates had virulent strain characteristics and other three have mesogenic characteristics. It was noticed that there was a mismatch in the indices of the MDT of the isolates. Previously a mismatch in the MDT and the ICPI values was observed [15].

## Figures and Tables

**Table 1:** The location of NDV outbreaks, year of isolation, type, breed, and age of birds involved.

Virus strain	Abbreviation	Location of outbreak	Year of isolation	Host	Type/breed of bird	Age
Layer/Nyala/08	LNy/08	Nyala	2008	Chicken	layer/lohman	18 weeks
Layer/Nyala/09	LNy/09	Nyala	2009	Chicken	layer/Bovans	15 months
Pigeon/Kamlen/09	Pkam/09	Kamleen	2009	Pigeon	—	Adult
Pigeon/Medni/010	Pmed/010	Medani	2010	Pigeon	—	Adult
Broiler/Halfaya/011	Bhalf/011	Halfaya	2011	Chicken	Broiler/Ross	30 days
Broiler/Soba/2013	Bso/013	Soba	2013	Chicken	Broiler/Cobb	6 days
Layer/Kabashi/2013	Lkab/013	Kabashi	2013	Chicken	layer/Hyline	6 months

**Table 2:** Haemagglutination pattern of the NDV field isolates to chicken, equine, sheep, and bovine RBCs.

NDV isolate	Chicken RBCs	Equine RBCs	Sheep RBCs	Bovine RBCs
LNy/08	6log_2_	0	0	0
LNy/09	10log_2_	5log_2_	0	0
Pkam/09	8log_2_	4log_2_	8log_2_	4log_2_
Pmed/010	5log_2_	0	0	0
Bhalf/011	10log_2_	0	0	0
Bso/013	7log_2_	4log_2_	6log_2_	3log_2_
Lkab/013	7log_2_	3log_2_	3log_2_	2log_2_

**Table 3:** Haemagglutination elution of the NDV field isolates.

NDV isolate	HA titre		
	1 hour	24 hours	30 hours
LNy/08	6log_2_	5log_2_	0
77	6log_2_	3log_2_	0
LNy/09	7log_2_	4log_2_	0
Pkam/09	6log_2_	4log_2_	0
Pmed/010	3log_2_	1log_2_	0
Bhalf/011	8log_2_	5log_2_	0
Bso/013	6log_2_	4log_2_	0
Lkab/013	6log_2_	5log_2_	0

**Table 4:** The ICPI values and the MDT produced by the NDV isolates.

NDV isolate	MTD^*^ (hours)	ICPI	Pathotype based on ICPI^**^
LNy/08	NT	1.3	Mesogenic
LNy/09	76.8	1.2	Mesogenic
Pkam/09	62.4	1.3	Mesogenic
Pmed/010	48	1.8	Velogenic
Bhalf/011	51	2.0	Velogenic
Bso/013	57	1.6	Velogenic
Lkab/013	54	1.6	Velogenic

^*^MDT: mean death time, measured in hours to death; ^**^ICPI: intracerebral pathogenicity index, based on an average score of clinical signs over time.
